# Supplementary material for: Practical blueprint for low-depth photonic quantum computing with quantum dots
Source: npj Quantum Inf. 2026 May 14;12(1):155. doi: 10.1038/s41534-026-01258-3 (PMC13616740; doi:10.1038/s41534-026-01258-3)
Supplement: Supplementary file 1 [file 41534_2026_1258_MOESM1_ESM.pdf]

Supplementary Material for "Practical blueprint for low-depth photonic quantum computing with quantum dots"

Ming Lai Chan[†,1,2,*] Aliki Anna Capatos,[3,4,†] Peter Lodahl,[1,2] Anders Søndberg Sørensen,[2] and Stefano Paesani[2,4,‡]

[1] *Sparrow Quantum, Blegdamsvej 104A, DK-2100 Copenhagen Ø, Denmark*
[2] *Center for Hybrid Quantum Networks (Hy-Q), The Niels Bohr Institute, University of Copenhagen, DK-2100 Copenhagen Ø, Denmark*
[3] *Quantum Engineering Centre for Doctoral Training, University of Bristol, Bristol, United Kingdom*
[4] *NNF Quantum Computing Programme, Niels Bohr Institute, University of Copenhagen, Blegdamsvej 17, DK-2100 Copenhagen Ø, Denmark*

(Dated: July 21, 2025)

I. STATE EVOLUTION IN THE PRESENCE OF ERRORS

In Sec. IV of the main text we considered two dominant imperfections during resource-state generation: branching errors due to finite optical cyclicity and laser-induced spin-flips during spin-rotation pulses. In this section, we illustrate how each of these errors affects the resource state produced by the time-bin protocol of Sec. III A 3. We begin in Sec. I A by reviewing the ideal, error-free evolution of a linear cluster state after one protocol round, which provides a baseline for comparison. Here we consider the linear cluster state for simplicity, but the principle applies equivalently to the encoded linear cluster state. We then introduce in Sec. I B the state evolution under branching errors, followed by spin-flip errors in Sec. I C.

A. Ideal state evolution

To establish a reference for error analysis, we first review the ideal, error-free evolution of the spin-photon system under two iterations of the protocol (subscripts and normalisation constant are omitted for brevity). The spin qubit is initialised in the ground state $|\uparrow\rangle$, and the protocol begins by preparing an equal superposition of spin states via a $\pi/2$ Hadamard rotation. This is followed by an optical early time-bin excitation, a spin π-rotation, another excitation for late time-bin, and another spin π-rotation. This completes one round of the time-bin protocol for a linear cluster state. To prepare an encoded qubit with N photons, the excitation-flip-excitation-flip steps are repeated $N-1$ times, before progressing to the next encoded qubit with another $\pi/2$ Hadamard. The ideal, error-free state evolution during the first round is described as follows:

(1)	*Prepare:*	$\lvert\uparrow\rangle + \lvert\downarrow\rangle$
	Excite:	$\lvert\uparrow\rangle + \lvert\downarrow\rangle \lvert e\rangle$
	π-Rotation:	$\lvert\downarrow\rangle - \lvert\uparrow\rangle \lvert e\rangle$
	Excite:	$\lvert\downarrow\rangle \lvert\ell\rangle - \lvert\uparrow\rangle \lvert e\rangle$
	π-Rotation:	$-\lvert\uparrow\rangle \lvert\ell\rangle - \lvert\downarrow\rangle \lvert e\rangle$
	Hadamard:	$-\lvert\uparrow\rangle \lvert\ell\rangle - \lvert\downarrow\rangle \lvert\ell\rangle - \lvert\uparrow\rangle \lvert e\rangle + \lvert\downarrow\rangle \lvert e\rangle \Rightarrow -\lvert\uparrow\rangle \lvert\ell\rangle - \lvert\uparrow\rangle \lvert e\rangle - \lvert\downarrow\rangle \lvert\ell\rangle + \lvert\downarrow\rangle \lvert e\rangle$

* Ming-Lai.Chan@sparrowquantum.com

† These authors contributed equally to this work.

‡ stefano.paesani@nbi.ku.dk

After a second round, this becomes:

(2)	*Excite:*	$-\|\uparrow\rangle\|\ell\rangle - \|\uparrow\rangle\|e\rangle - \|\downarrow\rangle\|\ell e\rangle + \|\downarrow\rangle\|ee\rangle$
	π-Rotation:	$-\|\downarrow\rangle\|\ell\rangle - \|\downarrow\rangle\|e\rangle + \|\uparrow\rangle\|\ell e\rangle - \|\uparrow\rangle\|ee\rangle$
	Excite:	$-\|\downarrow\rangle\|\ell\ell\rangle - \|\downarrow\rangle\|e\ell\rangle + \|\uparrow\rangle\|\ell e\rangle - \|\uparrow\rangle\|ee\rangle$
	π-Rotation:	$\|\uparrow\rangle\|\ell\ell\rangle + \|\uparrow\rangle\|e\ell\rangle + \|\downarrow\rangle\|\ell e\rangle - \|\downarrow\rangle\|ee\rangle$
	Hadamard:	$\|\uparrow\rangle\|\ell\ell\rangle + \|\downarrow\rangle\|\ell\ell\rangle + \|\uparrow\rangle\|\ell e\rangle + \|\downarrow\rangle\|\ell e\rangle + \|\uparrow\rangle\|e\ell\rangle - \|\downarrow\rangle\|e\ell\rangle + \|\uparrow\rangle\|ee\rangle - \|\downarrow\rangle\|ee\rangle$
		$\Rightarrow \|\uparrow\rangle\|\ell\ell\rangle + \|\uparrow\rangle\|\ell e\rangle + \|\uparrow\rangle\|e\ell\rangle + \|\uparrow\rangle\|ee\rangle + \|\downarrow\rangle\|\ell\ell\rangle + \|\downarrow\rangle\|\ell e\rangle - \|\downarrow\rangle\|e\ell\rangle - \|\downarrow\rangle\|ee\rangle$

In the last step of each round where the Hadamard is applied, the expression has been rearranged with the spin states grouped for clarity. The logic equally applies to encoded linear cluster states, where Hadamard gates are placed on every M-th round. The blue highlighted terms are those that can be affected by the dominant branching error mechanism. By comparison to this target state, we observe the effect on a resource state of a given noise source and deduce the equivalent Pauli error or loss that should then be modelled.

B. Branching error

We recall that the dominant branching error — an early photon that decays diagonally and is lost, followed by a correctly-emitted late photon — can only appear for the $|\uparrow\rangle$ state. If a branching error takes place in the first round, the resulting state before the Hadamard is given by:

(1)	*Prepare:*	$\|\uparrow\rangle + \|\downarrow\rangle$
	Excite:	$\|\uparrow\rangle - \|\uparrow\rangle\|\varnothing\rangle$ *branching*
	π-Rotation:	$\|\downarrow\rangle - \|\downarrow\rangle\|\varnothing\rangle$
	Excite:	$\|\downarrow\rangle\|\ell\rangle - \|\downarrow\rangle\|\varnothing\ell\rangle \Rightarrow \|\downarrow\rangle\|\ell\rangle - \|\downarrow\rangle\|\ell\rangle$
	π-Rotation:	$-\|\uparrow\rangle\|\ell\rangle + \|\uparrow\rangle\|\ell\rangle$

By comparing the blue term from the ideal round (1) case with the red term above, the effect of this branching error is an apparent X error applied to both the spin and photon respectively. However, since this is the first round and there is no previous spin-photon correlation to compare to, the result is solely an X error on the photon. Should branching occur in any subsequent n-th round instead, the effect is indeed both an X error on the spin and an X error on the nth-round photon. For branching error occurring during round (2), the state evolution is:

(2)	*After ideal round 1:*	$-\|\uparrow\rangle\|\ell\rangle - \|\downarrow\rangle\|e\rangle$
	Hadamard:	$-\|\uparrow\rangle\|\ell\rangle - \|\uparrow\rangle\|e\rangle - \|\downarrow\rangle\|\ell\rangle + \|\downarrow\rangle\|e\rangle$
	Excite:	$-\|\uparrow\rangle\|\ell\rangle - \|\uparrow\rangle\|e\rangle + \|\uparrow\rangle\|\ell\varnothing\rangle - \|\uparrow\rangle\|e\varnothing\rangle$ *branching*
	π-Rotation:	$-\|\downarrow\rangle\|\ell\rangle - \|\downarrow\rangle\|e\rangle + \|\downarrow\rangle\|\ell\varnothing\rangle - \|\downarrow\rangle\|e\varnothing\rangle$
	Excite:	$-\|\downarrow\rangle\|\ell\ell\rangle - \|\downarrow\rangle\|e\ell\rangle + \|\downarrow\rangle\|\ell\varnothing\ell\rangle - \|\downarrow\rangle\|e\varnothing\ell\rangle$
	π-Rotation:	$\|\uparrow\rangle\|\ell\ell\rangle + \|\uparrow\rangle\|e\ell\rangle - \|\uparrow\rangle\|\ell\varnothing\ell\rangle + \|\uparrow\rangle\|e\varnothing\ell\rangle \Rightarrow \|\uparrow\rangle\|\ell\ell\rangle + \|\uparrow\rangle\|e\ell\rangle - \|\uparrow\rangle\|\ell\ell\rangle + \|\uparrow\rangle\|e\ell\rangle$

Comparing these red terms with the blue ideal state terms after round (2) confirms the effect.

C. Laser-induced spin-flip effects

A π-pulse is applied between each time-bin, where a laser-driven spin-flip error may appear. Depending on the state of the spin when it experiences an imperfect rotation pulse, there are two possible outcomes [1]: If the spin starts in the $|\downarrow\rangle$ state, the rotation pulse fails to rotate it to the $|\uparrow\rangle$ state and there is zero photon emission in either time-bin. If the spin starts in $|\uparrow\rangle$, it will stay in this state for the late time-bin, resulting in an emitted photon in both time-bins respectively. Both possibilities lead to loss, since we either have no photon produced or double emission that takes the system state out of the computational space:

(1) *Prepare:* $|\uparrow\rangle + |\downarrow\rangle$

Excite: $|\uparrow\rangle + |\downarrow\rangle |e\rangle$

π-Rotation: $-|\uparrow\rangle - |\downarrow\rangle |e\rangle$ *(spin-flip error occurs)*

Excite: $-|\uparrow\rangle |\varnothing\rangle - |\downarrow\rangle |e\ell\rangle$

π-Rotation: $-|\downarrow\rangle |\varnothing\rangle + |\uparrow\rangle |e\ell\rangle \Rightarrow -|\downarrow\rangle + |\uparrow\rangle |e\ell\rangle$

In the final expression (highlighted in red), the $|\uparrow\rangle$ state shows no associated emission, and the $|\downarrow\rangle$ state does not produce a usable qubit. In addition to the spin flip and photon loss, we observe spin dephasing if the spin begins in the $|\uparrow\rangle$ state (an early time-bin photon) when comparing to the ideal state. Since the threshold for spin errors is lower than that for loss, we use the former as the limit for laser-induced spin-flips.

[1] Y. Meng, M. L. Chan, R. B. Nielsen, M. H. Appel, Z. Liu, Y. Wang, N. Bart, A. D. Wieck, A. Ludwig, L. Midolo, A. Tiranov, A. S. Sørensen, and P. Lodahl, Deterministic photon source of genuine three-qubit entanglement, Nature Communications **15**, 7774 (2024)
